# Positive Effect of 25-Hydroxyvitamin D (25(OH)D) Levels in Follicular Fluid on Embryonic Developmental Potential in Diminished Ovarian Reserve (DOR) Patients Undergoing Microstimulation

**DOI:** 10.7759/cureus.66436

**Published:** 2024-08-08

**Authors:** Huan Wang, Ling Yi, Jing Liu, Taifeng Mao, Wenyan Liu

**Affiliations:** 1 Department of Auxiliary Reproductive Health, Jiangxi Province Ji’an Women and Child Health Care Hospital, Ji’an, CHN

**Keywords:** in vitro fertilization, vitamin d, follicular fluid, microstimulation protocol, diminished ovarian reserve

## Abstract

Objective: This study aimed to explore the effect of 25-hydroxyvitamin D (25(OH)D) levels in follicular fluid (FF) on the embryo outcome with diminished ovarian reserve (DOR) patients undergoing in vitro fertilization (IVF) by microstimulation protocol.

Methods: A prospective cohort study of 79 patients with DOR who underwent IVF using the microstimulation protocol was conducted. Based on the level of 25(OH)D in follicular fluid (25(OH)D-FF), the patients were divided into a high-value group (25(OH)D-FF>11.1, n = 50) and a low-value group (25(OH)D-FF>11.1, n = 29). Correlation analysis was conducted between the level of 25(OH)D-FF and the rate of high-quality embryos on day 3 (D3). Logistic regression analysis of factors affecting the presence or absence of D3-available embryos in patients with DOR was conducted.

Results: The number of oocytes retrieved, mature oocytes (MII), normal fertilization rate, number of available embryos on D3, and high-quality embryo rate were lower in the low-value group than in the high-value group (p = 0.000, p = 0.000, p = 0.009, p = 0.000, p = 0.001). The clinical pregnancy rate of frozen embryo transfer (FET) between the two groups was no statistically significant difference (p > 0.05); correlation analysis between the 25(OH)D-FF level and the rate of high-quality embryos was performed using Spearman's rank-sum test, and there was a positive correlation (R = 0.271, P<0.01). Logistic analysis showed that 25(OH)D-FF level was a protective factor for embryonic outcome (odds ratio (OR) > 1, P<0.01).

Conclusion: The 25(OH)D level in FF has a positive effect on embryonic outcomes in DOR patients with IVF using the microstimulation protocol. Vitamin D (VD) supplementation can be used to increase the number of available embryos and improve the quality of embryos for patients with DOR who are undergoing microstimulation of IVF.

## Introduction

With the development of society, more and more women are delaying childbearing. The delay in childbearing age is accompanied by a decline in the number and quality of oocytes, that is age-related physiologic diminished ovarian reserve (DOR). Meanwhile, at the same time, with the aggravation of environmental pollution and social pressure, the incidence of age-unrelated non-physiological DOR also showed an increasing trend year by year. According to recent reports, the prevalence of DOR is about 10%-35% [1]. Although assisted reproductive technology (ART) has made great progress in the last two decades, the pregnancy rate of DOR patients is generally lower than 40% [2]. Therefore, how to improve the fertility of DOR patients and improve the quality of embryos is the current focus and difficulty in the field of reproduction.

Vitamin D (VD) is a steroid hormone that is mainly produced by the conversion of subcutaneous 7-dehydro-cholesterol under ultraviolet irradiation or oral intake and then hydroxylated to 25-hydroxyvitamin D (25(OH)D) by the liver. After hydroxylation by the kidney, it is converted into biologically active 1,23-dihydroxyvitamin D (1,25(OH)2D), and 1,25(OH)2D exerts its classical functions of regulating calcium-phosphorus homeostasis, bone metabolism, and maintenance of neuromuscularity by binding to the VD receptors (VDRs). Due to the short half-life of 1,25(OH)2D, deficient concentration in blood, and easy regulation by blood calcium-phosphorus feedback, 25(OH)D is commonly used in clinical assessment of VD levels in vivo.

Vitamin D exerts its biological effects by binding to VD receptors, and it is currently believed that VDRs are present in almost all cells or tissues of the human body, including human reproductive organs and tissues such as testes, breasts, ovaries, endometrium and placenta, hypothalamus, and pituitary gland [3]. Cell culture has shown that VD binds to its receptor and stimulates the production of estrone, estradiol, and androgen in the ovary [4]. In addition, basic studies have demonstrated that VD affects follicular growth and development by increasing cell proliferation [5] and inhibiting apoptosis and inflammatory processes in the follicle [5]. Animal experiments have shown that knockout of VDRs in mice resulted in blocked follicular development and impaired fertility [6]. Vitamin D deficiency not only affects bone health, muscle strength, autoimmune diseases, and type 2 diabetes [7]. Moreover, according to studies, VD deficiency may also be involved in the occurrence of primary ovarian dysfunction [8] and infertility [9].

Follicular fluid (FF) is where oocytes grow, develop, and eventually mature [10]. Various hormones and molecules in FF affect the growth and development of oocytes through autocrine or paracrine [11], and changes in the content of various hormones and molecules in the FF will directly affect the quality of oocytes and embryos [10]. Therefore, it is of great clinical significance to study the embryo outcome through FF.

A study conducted by Ekapatria et al. in Indonesia showed that oocyte quality was higher with high levels of 25 (OH) D in follicular fluid (25(OH)D-FF) [12]. Arnanz et al.'s study of FF from 226 patients from the Middle East showed that, in VD-deficient patients, the mean 25(OH)D-FF, free 25(OH)D, and bioavailable 25(OH)D concentrations were higher, the higher the probability of obtaining blastocyst haploid; however, no difference was found in non-deficient individuals [13]. Some other studies believe that the level of 25(OH)D-FF can well reflect the level of 25(OH)D in serum, and the embryo implantation rate and clinical pregnancy rate are higher in those with high 25(OH)D-FF [14]. On the contrary, some studies have suggested that there is no correlation between the two [15] or that there is a negative correlation between the two [16].

There is still much debate about whether VD levels in FF affect embryo outcomes in in vitro fertilization (IVF), and to fully understand the role of VD in DOR patients undergoing fertility treatments, this study prospectively explored the effect of 25(OH)D levels in FF on the embryonic outcome of patients with DOR undergoing IVF with microstimulation protocol.

## Materials and methods

Study design

The purpose and procedures of the study were explained to all included researchers before starting the study, and consent was obtained from all patients in the study. At the same time, the research program was approved by the ethics committee of our hospital, Jiangxi Province Ji’an Women and Child Health Care Hospital, in Ji'an, China (approval number: 2022A06120). All procedures were based on the 1964 Declaration of Helsinki and its subsequent amendments and research programs.

Patients

Data from 145 DOR patients undergoing IVF by microstimulation protocol from June 2021 to October 2023 in Jiangxi Province Ji’an Women and Child Health Care Hospital were included in the study. The study included patients aged between 18 and 45 years old, with a BMI of 16-30 kg/m^2^. The diagnostic criteria for DOR refer to the Expert Consensus on Clinical Diagnosis and Treatment of Diminished Ovarian Reserve [17], which are as follows: anti-Müllerian hormone (AMH) 0.5-1.1 ng/ml, basal follicle-stimulating hormone (FSH) 10 IU/L in two consecutive menstrual cycles, or antral follicle count (AFC)<5-7.

Exclusion criteria

Patients with a history of contraindications with assisted pregnancy, severe oligoasthenospermia and azoospermia, cryopreserved sperm, and a history of smoking and alcoholism use were excluded from the study.

Protocol

All patients received microstimulation to control ovarian hyperstimulation (COH). transvaginal ultrasound combined with gonadal hormones to monitor follicle growth; when one follicle's diameter exceeded 18 mm or two follicles' diameter exceeded 17 mm, serum levels of estradiol (E2), luteinizing hormone (LH), and progesterone (P) were measured on the same day, and 5,000 IU of human chorionic gonadotropin (HCG) was injected on the same night, and oocytes were retrieved after 36 hours of HCG injection. During oocyte retrieval, the FF from the first dominant follicle was retained. The oocytes obtained were inseminated and cultured in the laboratory by an embryologist, and the presence of two pronuclei and two polar bodies at 16-18 hours after fertilization confirmed that fertilization had occurred normally. All ovulation cycles were not fresh embryo transfer and whole embryo freezing. The FF obtained was determined by colorimetric assay; hemoglobin (Hb) > 1.0 g/L was considered to be contaminated with blood, and the sample failed.

Embryo assessment

The scoring of cleavage embryos was based on Peter's scoring criteria [18] into grades I-IV, of which grade I-II embryos were considered high-quality embryos, while grade I-III embryos were considered as available embryos.

Measurement of VD concentration

The obtained FF was centrifuged at 3,000 rpm, and 1 mL of the supernatant was retained and stored in a refrigerator at -80 °C. After collecting a sufficient sample volume, the samples were thawed uniformly, and 25(OH)D concentration was measured by enzyme-linked immunosorbent assay (ELISA) (EMABIO, Houston, TX).

Frozen embryo transfer (FET)

The patient underwent a hormone replacement cycle to prepare the endometrium. On day three to five of menstruation, transvaginal ultrasound was performed, and the patient was instructed to take oral estradiol valerate tablets (PEG, Bayer, Leverkusen, Germany) at 4-8 mg per day, and B-ultrasound monitoring of the endometrium started on day six of the medication, and the dosage of the drug was adjusted according to the thickness of the endometrium. After 10 days of the medication, the thickness of the endometrium reached the standard, and then the endometrial transformation was performed by giving a progesterone injection of 80 mg/d. The day of progesterone injection was day one, and the embryo transferred on day four.

The serum beta-hCG level was measured 14 days after the transfer to confirm whether it was pregnancy or not. Clinical pregnancy was diagnosed when a gestational sac and primitive cardiac tube pulsation were seen in the uterine cavity by transvaginal ultrasound 28 days after transfer.

Statistical methods

Statistical analysis was performed using IBM SPSS Statistics software for Windows, version 21 (IBM Corp., Armonk, NY). The Kolmogorov-Smirnov method was used to test the normality of all data, and none of the measurements in this study were normally distributed. Continuous variables were expressed as the median (25^th^ percentile, 75^th^ percentile) (M(P25, P75)), and the Mann-Whitney U test was used for comparison between groups. categorical variables were expressed as a rate (%), using the continuity correction Chi-square test or Fisher exact test. Spearman's rank correlation coefficient (rS) was used for correlation analysis. Logistic regression was used to identify independent variables associated with D3-available embryos, and the results interpretation criteria were: odds ratio (OR) >1 as a protective factor, OR<1 as a risk factor. The significance level for all tests was set at P<0.05.

## Results

**Figure 1:**
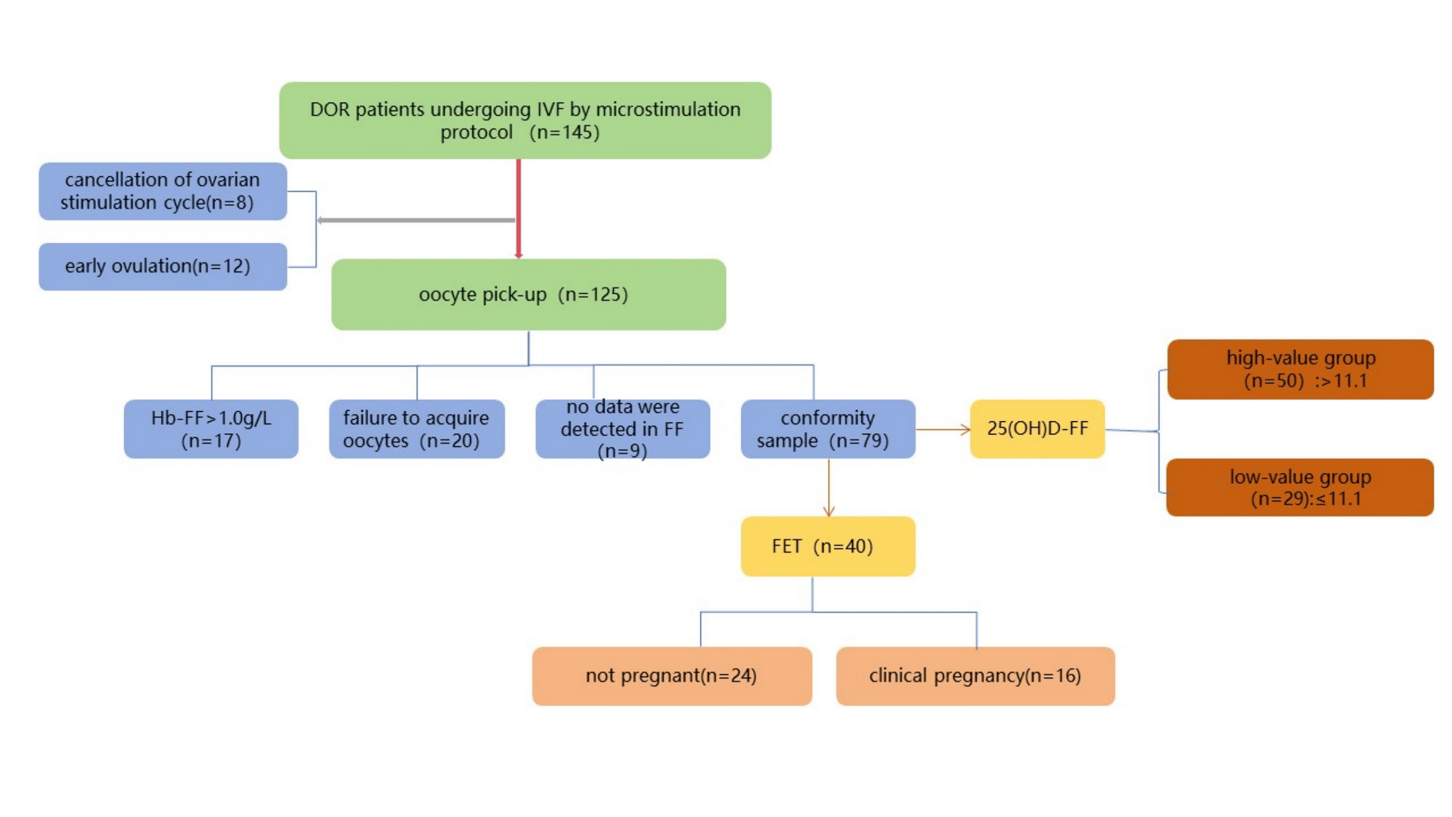
Flowchart of the experimental design DOR: diminished ovarian reserve; IVF: in vitro fertilization; Hb-FF: hemoglobin in follicular fluid; 25(OH)D-FF: 25-hydroxyvitamin D in follicular fluid; FET: frozen embryo transfers

As shown in Figure 1, microstimulation protocol 145 cases, of which eight of the DOR patients with microstimulation canceled ovulation promotion for various reasons; 12 canceled oocyte retrieval due to premature follicular ovulation. A total of 125 patients collected FF samples, of which 20 cases without oocytes were retrieved and 17 cases with Hb>1.0 g/L. No data were detected in FF in nine cases. A total of 79 patients were included in the statistical analysis, which included 40 cases of FET. With or without the number of available embryos on day three as the target, the cut-off value of 25(OH)D-FF was 11.10 ng/mL, the sensitivity was 73.2%, and the specificity was 60.9%. According to this, the 25(OH)D-FF was divided into a high-value group (>11.1 ng/mL) and a low-value group(≤11.1ng/mL).

As shown in Table 1, basal characteristics, stimulation parameters, and clinical pregnancy rate of FET were similar between the comparison groups. The AMH (p = 0.000), number of oocytes retrieved (p = 0.000), mature oocytes (MII) (p = 0.000), normal fertilization rate (p = 0.009), number of available embryos on day three (p = 0.000), and high-quality embryo rate (p = 0.001) in the low-value group were significantly lower than those in the high-value group.

**Table 1:** Participant and assisted reproductive technology cycle characteristics by 25(OH)D-FF 25(OH)D-FF: 25-hydroxyvitamin D in follicular fluid; BMI: body mass index; AMH: anti-Müllerian hormone; FSH: follicle-stimulating hormone; LH: luteinizing hormone; E2: estradiol; p: progesterone; MII: mature oocyte; FET: frozen embryo transfer; P <0.05 was considered significant.

	Low-value group	High-value group	X^2^/Z	P-value
Number of patients (n)	29	50		
Female age (years)	39.0（36.0，42.0）	40.0（36.0，42.0）	-0.951	0.342
Male age (years)	40.0（36.5，44.0）	40.0（38.0，42.0）	-0.833	0.405
BMI (kg/m^2^)	21.60（19.98，22.43）	21.75（20.60，22.60）	-1.115	0.265
AMH (ng/mL)	0.33（0.11，0.47）	0.49（0.31，0.88）	-5.124	0.000*
Basal FSH (mIU/ml)	10.79（8.48，13.63）	10.66（7.35，13.8）	-.345	0.730
Basal LH (mIU/ml)	4.83（3.03，5.61）	4.735（3.53，5.94）	-.770	0.441
Basal E2 (pg/ml)	36.38（22.46，56.56）	39.79（30.91，61.33）	-2.327	0.244
Sperm DNA fragmentation rate (%)	20.00（11.35，25.92）	19.35（11.62，33.73）	-0.641	0.522
Hemoglobin (g/L)	0.55(0.38,0.70)	0.60(0.45,0.80)	-1.119	0.263
Sperm forward motility rate (%)	27.00（20.70，37.50）	33.50（27.50，40.25）	-1.726	0.084
LH level on trigger day (mIU/ml)	8.43（6.91，10.52）	8.77（6.39，11.37）	-.168	0.867
E2 level on trigger day (pg/ml)	556.8（439.2，975.5）	682.15（496.9，1007.2）	-1.902	0.057
P level on trigger day (ng/mL)	0.27（0.21，0.40）	0.30（0.20，0.44）	-1.053	0.292
Number of oocytes retrieved (n)	1（1，2）	2（1，2）	-5.067	0.000*
MII oocytes (n)	1（1，1）	1.5（1，2）	-3.870	0.000*
Fertilization rate (%)	0（0，0）	0（0，1）	-2.611	0.009*
Number of available embryos on day 3 (n)	1（0，1）	1（1，2）	-5.512	0.000*
High-quality embryo rate on day 3 (%)	0（0，0）	0（0，0）	-3.355	0.001*
Clinical pregnancy rate of FET (%)	33.33(4/12)	42.86%(12/28)		0.833

**Figure 2:**
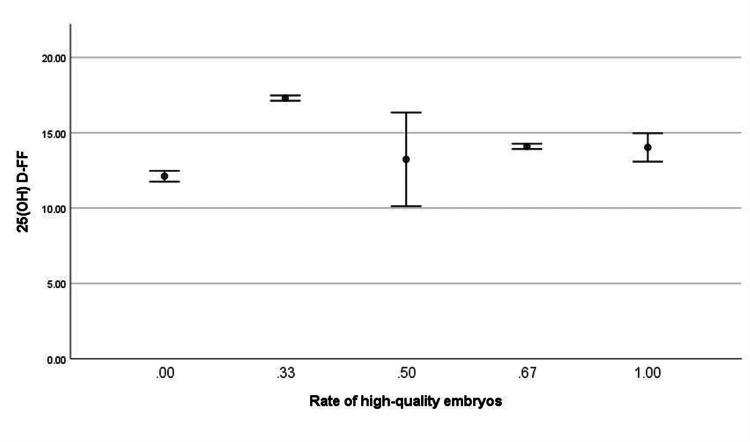
Correlation analysis 25(OH)D-FF: 25-hydroxyvitamin D in follicular fluid

As shown in Figure 2, the correlation between 25(OH)D-FF levels and the rate of high-quality embryos was analyzed using Spearman's rank sum test, and there was a statistically weak positive correlation between the two (R = 0.271, P <0.01).

**Figure 3:**
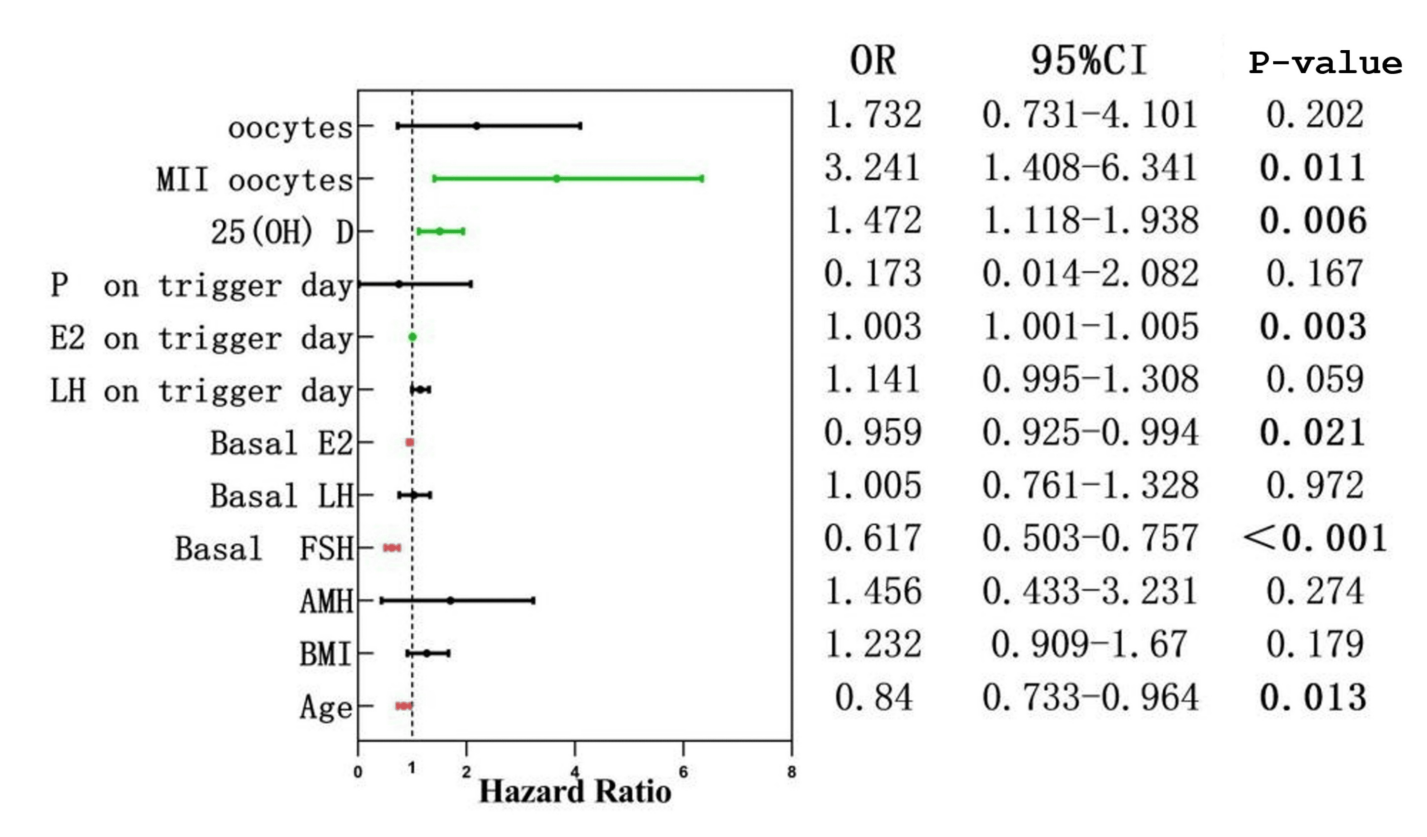
Logistic regression OR: odds ratio; MII: mature oocyte; 25(OH)D: 25-hydroxyvitamin D; p: progesterone; E2: estradiol; LH: luteinizing hormone; FSH: follicle-stimulating hormone; AMH: anti-Müllerian hormone; BMI: body mass index; P<0.05 was considered to be statistically significant.

As shown in Figure 3, the logistic regression model includes the number of available embryos on day three as dependent variables; female age (OR: 0.84; P = 0.013), basal FSH (OR: 0.617; P = 0.001), and basal E2 (OR: 0.959; P = 0.021) were risk factors for the formation of available embryos, whereas E2 level on trigger day (OR: 1.003; P = 0.003), 25(OH)D-FF level (OR: 1.472; P = 0.006), and MII oocyte number (OR: 3.241; P = 0.011) were protective factors for the formation of available embryos. 

## Discussion

There are no standardized criteria for 25(OH)D levels in follicular fluid, the main reference being the Italian Association of Clinical Endocrinologists (AME) in conjunction with the Italian Chapter of the American Association of Clinical Endocrinologists (AACE), which published a statement on the clinical management of vitamin D deficiency in adults in 2018, where 25(OH)D less than 20 ng/mL (50 nmol/L) diagnoses a deficiency [19]. Our study aimed to explore the correlation between 25(OH)D levels in FF and the embryo outcome in DOR patients undergoing IVF with microstimulation protocol. We finally included 79 patients with FF samples for statistical analysis. The results of 25(OH)D-FF testing revealed that the results were all at deficient levels. This is consistent with previous findings that VD is generally deficient in Chinese women [20], which may be related to less outdoor exercise, lack of sunlight exposure, and air pollution. At the same time, approximately 30%-60% of children and adults worldwide have VD deficiency and insufficiency [21]. Vitamin D deficiency has become one of the important public health problems in our country and even globally.

The AMH is a dimeric glycoprotein produced by granulosa cells of the preantral and small antral follicles, which inhibits follicle recruitment and growth and promotes the selection of dominant follicles [22]. Skowronska et al. showed that there was a positive correlation between FF VD level and AMH [23]. Ana et al.'s study on the relationship between FF VD and blastocyst euploidy found that VD level in patients with vitamin D deficiency was positively correlated with AMH, and was independent of age [13]. A study showed that low levels of VD may lead to a decrease in ovarian reserve, which may be related to the high degree of similarity between the VD response element and the AMH gene promoter [24]. All patients in this study were DOR patients with low AMH levels; the results supported the previous conclusion. The results of this study showed that AMH in the group with high VD levels was higher than that in the group with low VD levels (P<0.001). However, the results of logistic regression analysis showed that AMH was not an independent influence on D3-available embryos; we speculate that VD may influence D3-available embryos through AMH.

Interestingly, our results showed that VD level was positively correlated with the rate of D3 high-quality embryos, which was consistent with previous research results [13]. With the presence of available embryos at D3 in patients with DOR as the dependent variable, logistic regression analysis was performed. The results showed that the E2 level on trigger day, 25(OH)D-FF level, and the number of MII oocytes were protective factors for the formation of available embryos. The results showed that the level of 25(OH)D-FF was positively correlated with the quality of D3 embryos in patients with DOR undergoing microstimulation.

The positive correlation between VD and D3 embryo quality may be related to the regulatory effect of VD on AMH and ovarian steroid hormones. Previous studies [25] have shown that the AMH gene promoter has a VD receptor, and AMH is positively affected by VD. This is consistent with the conclusion of this study that the AMH of the high-value group is higher than that of the low-value group. Under physiological conditions, AMH acts on anti-Müllerian hormone receptor II (AMHR-II) to inhibit follicle recruitment and granulosa cell differentiation, while VD can inhibit AMHR-II, thus weakening the inhibitory effect of AMH on follicle recruitment and granulosa cell differentiation, resulting in more follicles eventually growing and maturing [26]. At the same time, Nandi et al. [25] showed that there were VD response elements on the promoter of key enzyme genes that catalyzed the production of estradiol and progesterone, therefore, VD deficiency may interfere with steroid hormone production, follicular development, and oocyte maturation [14].

The positive effect of VD on the quality of D3 embryos might also be explained by the antioxidant effect of VD. The presence of reactive oxygen species (ROS) and reactive nitrogen inside and outside the cell, these reactive molecules can trigger oxidative reactions in the cell, causing cell and tissue damage. It has been reported that supraphysiologic amounts of ROS are associated with embryonic developmental defects [27]. However, the antioxidant enzyme systems present in the body, such as superoxide dismutase (SOD), catalase (CAT), and selenium-dependent glutathione peroxidase (SeGPx) systems, work together to prevent free radicals from damaging cellular tissues. Olszak-Wąsik et al. [28] demonstrated that glutathione peroxidase (GPX) in the FF was positively correlated with the quality of the embryo and the pregnancy rate; meanwhile, Paszkowski concluded that high levels of SeGPx in the FF have a positive effect on fertilization success [29]. Vitamin D protects gamete growth and development by upregulating the expression of antioxidative enzymes and reducing oxidative stress [30]. In 2013, Jain and Micinski found that VD supplementation upregulated U937 monocyte cell expression of glutamate cysteine ligase (GCLC) and glutathione reductase (GR) to increase cellular glutathione (GSH) levels and inhibited ROS and secretion of the pro-inflammatory cytokines [30]. Therefore, the improvement of embryo quality by VD supplementation may be mediated by upregulating the expression of antioxidant enzymes, increasing cellular GSH levels, and reducing oxidative stress.

There was no difference in the clinical pregnancy rate between the two groups after subsequent freezing and thawing, which is related to the fact that clinical pregnancy is not only related to the quality of the embryos but also the endometrium, the maternal environment, and the freezing and thawing techniques, among other factors.

The limitation of this study is that serum 25(OH)D levels were not tested, and therefore serum vitamin D data could not be utilized to classify FF vitamin D. Another limitation is that the normal value of 25(OH)D in follicles was not known. Thus, the cutoff value for further categorization of the low and high FF vitamin D level groups was based on the number of subjects with and without D3-available embryos. At the same time, this study was a single-center study with a limited sample size, and the results obtained may be biased. Therefore, a larger sample size and a multicenter study are needed to obtain more realistic and reliable data.

## Conclusions

To the best of our knowledge, this is the first study to investigate the relationship between VD and embryonic outcomes in DOR patients using microstimulation protocols, and this study demonstrates that VD has a positive effect on the embryonic outcomes of patients with DOR, with a significantly higher chance of obtaining usable and high-quality embryos in patients with high VD levels. Therefore, in patients with DOR, appropriate VD supplementation can be used to increase the number of available embryos and improve the quality of embryos for subsequent pregnancy outcomes.
